# Epileptic patients’ willingness to receive cell-phone based medication reminder in Northwest Ethiopia

**DOI:** 10.1186/s12911-019-0830-z

**Published:** 2019-06-24

**Authors:** Bereket Senay, Kassahun Dessie Gashu, Adamu Takele Jemere, Zeleke Abebaw Mekonnen

**Affiliations:** 1 University of Gondar Comprehensive Specialized Hospital, Gondar, Ethiopia; 2 Department of Health Informatics, Institute of Public Health, College of Medicine and Health Sciences, University of Gondar, Gondar, Ethiopia

**Keywords:** Cell-phone medication reminder, Epilepsy, Medication adherence, Ethiopia

## Abstract

**Background:**

Poor adherence compromises medication treatment effectiveness which results in suboptimal illness control. This can lead to increased use of healthcare services, reduction in patients’ quality of life and increased healthcare costs. Reminding patients of their medication intake increases their adherence. Therefore, this study aimed to assess the willingness of epileptic patients to receive cell-phone based medication reminders and its associated factors in Northwest Ethiopia.

**Methods:**

Institution based cross sectional study was conducted in the study period scheduled from March 1 to April 30, 2018 to interview 422 study participants at University of Gondar Comprehensive and Specialized Hospital, Northwest Ethiopia. Systematic random sampling was used to select 422 epileptic patients. A structured interviewer administered questionnaire was used to collect data and analyzed by using SPSS version 21. Binary and multivariate logistic regression analysis was performed to identify the determinant factors for willingness to receive cell-phone based medication reminders. *P* < 0.05 at 95% confidence interval was considered statistically significant.

**Results:**

A total of 394 (93% response rate) respondents were interviewed. The majority of respondents 262 (66.5%) owned a cellphone. Among the participants 271 (68.8%) were willing to receive reminder messages. In the multivariate regression analysis; living in urban areas (AOR = 5.63, 95% CI; 3.18–9.96), experience of forgetting things (AOR = 2.63, 95% CI; 1.44–4.80), forgetting to take Long-term Antiepileptic Drugs (AEDs) (AOR = 2.17, 95% CI; 1.06–4.43) and average monthly income ≥2000 birr (AOR = 2.43, 95% CI; 1.03–5.75) were significantly associated with willingness to receive cell-phone medication reminders. Pertaining to marital status; being married (AOR = 5.75, 95% CI; 1.11–29.70) or divorced (AOR = 5.15, 95% CI; 1.29–20.49) participants were also more willing to receive cell-phone medication reminders as compared to singles.

**Conclusion:**

Most respondents have a cellphone and were willing to use it as a medication reminder. Marital status, place of residence, average monthly income, experience of forgetting things since they started AED, forget to take AED are the most notable factors that are associated with the willingness of patients to receive cell phone drug reminder messages.

## Background

Epilepsy is a chronic disorder of the brain that affects people of all ages, is one of the world’s oldest recognized conditions, dating over 4000 years of ignorance, superstition and stigmatization [1]. Birth trauma, CNS infections, cerebrovascular disease, cerebral tumors, febrile convulsions, toxins, genetic predisposition, metabolic disease and traumatic brain injury are the most commonly implicated causes of Epilepsy [2]. Epileptic patients have 3 fold higher risk of premature death where the problem is rampant in developing countries particularly in rural areas [3].

Worldwide, Epilepsy affects about 70 million people of all ages. The disease contributes 1 % to the global burden of which 80% is in low and middle-income countries [4]. Overall prevalence of Epilepsy is estimated to be 10 per 1000 people worldwide [5]. In Africa, the estimated prevalence ranges from 2.2 to 58 per 1000 people [6, 7]. In Ethiopia, the estimated incidence of Epilepsy is 64 per 100,000 people [8].

AEDs, which remain the main stay of Epilepsy treatment, reduce seizure frequency in up to 67% of epileptic patients [9]**.** The quality of life of epileptic patients could be significantly improved by adherence to AEDs which involves patients to take their medications as prescribed with respect to the dosage and dosage intervals [10]**.**

Management for chronic diseases like Epilepsy requires patients to take complex medication regimens on daily basis. Managing medication schedules may pose a significant burden in patients’ lives [11]**.** In Ethiopian context epileptic patients collect their medications on schedule bases weekly and monthly depending on their adherence level. In the mean time they are supposed to take their daily medications routinely for which they need a minimum of one daily reminder. Therefore, interventions, like medication reminders are found to be promising in improving patients’ adherence. Medication reminders can offer a mechanism to reduce unintentional non-adherence [12]**.**

An electronic health service readiness assessment study in Ethiopia indicated that the country’s increasing growth of mobile network coverage and affordability of mobile phones by low income inhabitants makes mobile-based health services are feasible for consultation, creating awareness, diagnosis and treatment [13]**.** The global coverage of wireless technology reached 96% where there are 6.8 billion mobile subscribers [14]**.** Mobile cellular telephone subscription rates of many African countries have reached more than 30% (world average is ≈ 78%) [15]**.** Sub-Saharan Africa has registered the highest cell phone uptake rate in the world with rapidly expanding mobile network coverage rate [16]**.** In Ethiopia, there were 57.34 million mobile subscribers at 2017 [17] and mobile network coverage was 90% [18]**.**

Even though educating patients to strictly follow medication regimens is key to Epilepsy treatment, non-adherence may interfere with seizure control. Many patients, mainly those with chronic illnesses, experience difficulties in treatment adherence. Non-adherence to medication treatment regimens is a worldwide health problem. Non-adherence rates among adults with Epilepsy ranges from 29 to 39% [19]**.**

Poor adherence compromises medication treatment effectiveness which results in suboptimal illness control. This can lead to increased use of healthcare services, reduction in patients’ quality of life, and increased healthcare cost [20–22]. Clinicians treating patients with Epilepsy noted that non-adherent patients report more difficulty in attaining seizure control compared to adherent patients. Uncontrolled seizures lead to major morbidity and mortality, including not only physical injury (head trauma, fractures and burns) but also psychosocial problems (depression, anxiety disorders, decreased quality of life, and sudden unexpected death) [23]**.** The increased risk of sudden unexplained death in epileptic patients is the most serious consequence of AED non-adherence [24]**.**

Forgetfulness is the common reported barrier to adherence in various patients. Problems with cognition are common in patients with Epilepsy. The International Bureau for Epilepsy survey revealed that 44% of patients with Epilepsy complained difficulty of learning, 45% felt that they were slow thinkers, 59% felt sleepy or tired, and 63% AED effects prevented them from achieving activities or goals [25]**.** Cognitive deficits could compromise medication adherence, there need to be a way to remind patients of their medication intake like reminding them through their cell phones. The ever-growing mobile telecommunication technology creates a great opportunity to implement an automated cell-phone based medication reminder systems to improve patient’s adherence to their medication. Hence, the purpose of this study is to assess the willingness and associated factors of epileptic patients to use mobile reminder systems.

## Methods

### Study area and period

The study was conducted at University of Gondar Comprehensive Specialized Hospital Neurology Center from March 1 to April 302,018. It’s located in Gondar Town, Northwest Ethiopia. The hospital is servicing for wider catchment population in Northwest Ethiopia.

### Study design and population

Institution based cross-sectional study was deployed to identify factors associated with willingness to receive cellphone based Medication reminder among epileptic patients. Patients who own cellphone were directly asked for their willingness to receive Medication reminder. Whereas, a case scenario was prepared for epileptic patients who don’t have cellphone couldn’t know about techniques of a mobile phone. The scenario was presented on a textbox below:

*“Mobile phone is a small electronic handheld device which can be easily put in a pocket and mainly used to communicate persons who are or not at a nearby. Mobile phone has many functions. Among them alarm function is a commonly mentioned strategy for remembering to take medications on time. A regularly received text or voice messages from treatment supporters is an alarm type that reminds patients to take medications on time.”*

The case scenario was used to give clue on the technic and purpose of the reminder. A case scenario was used to reduce response bias due to difference information about cellphone between mobile owners and non-owner patients.

The study population was all epileptic patients who had follow-up during data collection period at study area. Epileptic patients who couldn’t take medications by themselves (mentally ill, children and old aged patients) were excluded from the study.

### Sample size and sampling techniques

The desired sample size was calculated using single population proportion formula using assumptions; proportion of willingness (P) = 50%, margin of error (d) = 5 and 10% for non-response rate; the maximum sample size for this study was, *n* = 422 epileptic patients. Study participants were selected by systematic random sampling technique. On average 40 epileptic patients attend treatment follow-up at Neurology center per day. Taking 40 attendants per day and considering 44 days of data collection, a total of 1760 epileptic patients were used as a sampling frame to calculate the sampling interval. Thus, by dividing the sampling frame to the sample size, every 4th epileptic patient as per their order of visit was interviewed.

### Data collection tools and procedures

The dependent variable of the study was willingness to receive cell-phone based medication reminder. The independent variables include; socio-demographic factors, behavioral factors, psychosocial factors, environmental factors and patient provider relationship. Access and patterns of cell phone use were assessed as follows:

Patients’ access to cellphone was assessed using questions like: do you have a cellphone? Do you use more than one phone? Do you share cellphone with others? And how often do you have your cellphone with you?

Cellphone utilization were assessed using questions including: Do you have a cellphone? How often do you have your cellphone with you? Do you share cellphone with others? Did your cellphone, damaged, lost or stolen in the past? Do you switch off cellphone? Are there times or places where no calls are answered? Do you use text messages service with cellphone? How often your text messages seen by others? Do you use internet with cellphone? And what is your preferred way of cellphone communication?

Willingness to receive cellphone Medication reminder was measured by asking a question ‘Are you willing to receive medication reminder messages with your phone?’; a patients who answers ‘yes’ were considered as willing and those who says ‘no’ weren’t.

Data were collected using structured interviewer administered questionnaires. The questionnaire was primarily prepared in English and translated in to local language (Amharic) and back translated to English to check its consistency. Pretest was conducted at the nearby district hospital Debark Hospital with 21 participants (5% of the total sample size). A 1 day training was given for data collectors (three nurses) who were recruited to interview the study participants. The investigator conducted daily supportive supervision during data collection.

### Data processing and analysis

Data were entered using Epi-Info version 7 and exported to SPSS version 21 for analysis. Descriptive statistics were performed to describe the study population in relation to relevant variables. A bi-variable and multi-variable binary logistic regression analysis was done to identify factors significantly associated with the response variable. The association between the independent variables and the outcome variable was assessed using AOR and 95% CI and *P*-value less than 0.05 as cut-off point for statistical significance.

## Results

### Socio-demographic characteristics

A total of 394 epileptic patients attending follow-up at University of Gondar Comprehensive Specialized Hospital were included in the study (93% response rate). Most of the study participants were males 245 (62.2%) and about 109 (27.7%) of the participants were in the age range of 31–40 years; the mean age being 32.2 (±11.4) years. About 182 (46.2%) of them were married and 117 (29.7%) of them were illiterates. The average monthly income of 127 (32.2%) of the participants were less than 500 birr and almost half of the respondents 207 (52.5%) were rural settlers (Table 1).

**Table 1:** Socio-demographic characteristics of participants at University of Gondar Comprehensive and Specialized Hospital, Northwest Ethiopia, 2018 (*n* = 394)

Socio-demographic Characteristic	Category	Frequency	Percent
Sex	Male	245	62.2
	Female	149	37.8
Age (years)	16–24	104	26.4
	25–30	103	26.1
	31–40	109	27.7
	> 40	78	19.8
Marital status	Single	166	42.1
	Married	182	46.2
	Widowed	21	5.3
	Divorced	25	6.3
Religion	Orthodox	335	85.0
	Muslim	39	9.9
	Other	20	5.1
Educational status	Illiterate	117	29.7
	Able to read and write	31	7.9
	Primary	99	25.1
	Secondary	68	17.3
	Diploma and above	79	20.1
Occupation	Daily laborer	14	3.6
	Employed	140	35.5
	Student	68	17.3
	Merchant	32	8.1
	Farmer	77	19.5
	House wife	48	12.2
	Other	15	3.8
Residence	Urban	187	47.5
	Rural	207	52.5
Average monthly income (Birr)	< 500	94	23.9
	501–1000	127	32.2
	1001–2000	102	25.9
	> 2001	71	18.0

### Cellphone ownership and pattern of use

About 262 (66.5%) of the study participants owned a cellphone. Among those who owned a cellphone, 19.9% (*n* = 52) had more than one cellphone and 149 (56.9%) always had their cellphone with them. About 152 (58.0%) and 203 (77.5%) of them were able to use internet and text messaging service on their cellphone respectively. Most of the study participants 226 (86.2%) text messages are open to be seen by others (Table 2).

**Table 2:** Ownership and patterns of cellphone use among Epileptic patients at University of Gondar Comprehensive and Specialized Hospital, Northwest Ethiopia, 2018 (*N* = 262)

Variables	Category	Frequency	Percent
Own more than one cell phone	Yes	52	19.9
	No	210	80.15
Have cell phone with them	Always	149	56.9
	Most of the time	78	29.8
	Sometimes	35	13.3
Share cellphone with others	Yes	30	11.5
	No	232	88.5
Cellphone damaged, lost or stolen in the past	Yes	89	34.0
	No	173	66.0
Switch off cellphone	Yes	119	45.4
	No	143	54.6
Don’t answer calls in some places or times	Yes	138	52.7
	No	124	47.3
Can use text messaging service	Yes	203	77.5
	No	59	22.5
Text messages can be seen by others	Always	42	16.0
	Most of the time	82	31.3
	Sometimes	102	38.9
	Never	36	13.8
Use internet with mobile phone	Yes	152	58.0
	No	110	42.0

### Cell-phone based medication reminder

About 74 (18.8%) of the study participants were able to take their medication without a reminder. On the other hand, 14 (3.6%) and 48 (12.2%) of the study participants used their cellphone and other means as a reminder to take their medication respectively. Most of the participants 271 (68.8%) were willing to receive reminder messages to help them take their medication and almost half of them 202 (51.3%) were willing to pay for the medication reminder messages. Some of the respondents 26 (6.6%) believed that reminder massages lacks confidentiality (Table 3).

**Table 3:** medication reminder related characteristics of participants at University of Gondar Comprehensive and Specialized Hospital, Northwest Ethiopia, 2018 (*n* = 394)

Medication reminder related characteristics	Category	Number	Percent
Ever used any kind of medication reminder	Yes	48	12.2
	No	346	87.8
Already using cellphone based medication reminder	Yes	14	3.6
	No	380	96.4
Willing to receive cellphone based medication reminder	Yes	271	68.8
	No	123	31.2
Preferred type of cellphone communication	Voice call	121	30.7
	Short message service	131	33.2
	Email	20	5.1
Willing to pay for medication reminder messages	Yes	202	51.3
	No	70	17.8
Feel that reminder messages lack confidentiality	Yes	26	6.6
	No	368	93.4

### Factors associated with willingness to receive cellphone based AED reminder

A binary logistic regression analysis was done for every explanatory variable to include into the final multivariable logistic regression model. Then the variables with *p*-value of less than 0.2 were included into the final model and association of the explanatory variables with willingness to receive medication reminder messages was assessed. Place of residence were found to be a strong determinant factor for willingness to receive medication reminder. Urban residents were found to be 5.63 times more likely to receive medication reminder messages than rural settlers (AOR = 5.63, 95% CI; 3.18–9.96). Experience of forgetting things (AOR = 2.63, 95% CI; 1.44–4.80) and forgetting to take AEDs were also significantly associated with willingness to receive medication reminders (AOR = 2.17, 95% CI; 1.06–4.43). Smoking, missing to take a dose of AED and attending a health care appointment were found to be a weaker determinant factor for willingness to receive medication reminder. Higher average monthly income had also contributed to willingness of the participants to receive a medication reminder. The study had shown a significant relationship (AOR = 2.43, 95% CI; 1.03–5.75) between average monthly income and willingness to receive medication reminder (< 500 birr vs ≥ 2000 birr). Being married or divorced were also found to be significant determinant factors for willing to receive medication reminder (Table 4).

**Table 4:** Factors associated with willingness to receive cellphone based medication reminder for AED at University of Gondar Comprehensive and Specialized Hospital, Northwest Ethiopia, 2018 (*n* = 394)

Variable	Category	Willingness		OR (95%CI)	AOR (95%CI)
		Yes, *n* (%)	No, *n* (%)		
Marital status	Single	103 (62.0)	63 (38.0)	1.00	1.00
	Married	133 (73.1)	49 (26.9)	1.66 (1.06–2.61)	5.75 (1.11–29.70)*
	Widowed	13 (61.9)	8 (38.1)	0.99 (0.39–2.53)	3.98 (1.00–15.77)
	Divorced	22 (88.0)	3 (12.0)	4.49 (1.29–15.60)	5.15 (1.29–20.49)*
Residence	Urban	160 (85.6)	27 (14.4)	5.13 (3.14–8.37)	5.63 (3.18–9.96)*
	Rural	111 (53.6)	96 (46.4)	1.00	1.00
Monthly income	< 500	47 (50.0)	47 (50.0)	1.00	1.00
	500–1000	86 (67.7)	41 (32.3)	2.10 (1.21–3.63)	0.86 (0.36–2.06)
	1001–2000	81 (79.4)	21 (20.6)	3.86 (2.06–7.22)	0.85 (0.38–1.94)
	> 2000	57 (80.3)	14 (19.7)	4.07 (2.00–8.29)	2.43 (1.03–5.75)*
Smoking	Yes	18 (94.7)	1 (5.3)	8.68 (1.15 65.77)	3.16 (0.37–26.81)
	No	253 (67.5)	122 (32.5)	1.00	1.00
Experience forgetting	Yes	169 (80.9)	40 (19.1)	3.44 (2.19–5.39)	2.63 (1.44–4.80)*
	No	102 (55.1)	83 (44.9)	1.00	1.00
Missed Clinic appointment	Yes	131 (78.9)	35 (21.1)	2.35 (1.49–3.72)	1.16 (0.60–2.25)
	No	140 (61.4)	88 (38.6)	1.00	1.00
Ever missed a dose of AED	Yes	174 (74.7)	59 (25.3)	1.95 (1.26–3.00)	1.24 (0.62–2.47)
	No	97 (60.2)	64 (39.8)	1.00	1.00
Ever Forget to take AED	Yes	101 (82.1)	22 (17.9)	2.73 (1.62–4.60)	2.17 (1.06–4.43)*
	No	170 (62.7)	101 (37.3)	1.00	1.00

*AOR with *p*-value < 0.05

## Discussion

The results of the present study showed that about 262 (65.5%) of the study participants had owned a cellphone; of which 33 (12.6%) of them already uses the alarm function of their cell phone as a medication reminder. A study conducted in the same institution on willingness to receive text message medication reminders among patients on antiretroviral treatment, indicated that about 76.1% of the respondents had a personal cell phone. Among them, 71.2% uses their cell phone as a medication reminder [26]. This difference might be due to the place of residence of most of the study participants in this study; more than half of them were rural residents.

In this study, about 271 (68.8%) of the total respondents and 252 (96.2%) of cell phone owners in particular were willing to receive either a voice call, text or both. In addition, for 203 (77.5%) of them text messaging service was the most preferred way to receive a medication reminder. Different studies also reported more or less similar findings; a study on RVI patients in University of Gondar Hospital; almost all respondents 303 (95.9%) who owned a cell phone were willing to be contacted by the health institution through their cell phone; of which text message was the preferred means [26]. Studies in in South Africa, rural India and UK also supported text messaging as most preferred reminder [27–29]. This could be due to the feasibility of text than voice messaging, to be easily used by both sender and receiver.

Different reasons associated with non-willingness of patients to receive medication reminder messages were identified by different studies. In this study, be able to take their medication without a reminder and lack of trust in the confidentiality of reminder messages were the main reasons mentioned among those participants who were not willing to receive medication reminders. In line with this result, a study on RVI patients at University of Gondar reported that some of non-willing patients believed that text messages written about their medication would ruin their privacy [26]. In another study, those respondents who refused reported that they remembered to take their medication without reminders [28].

Studies indicated that treatment adherence to antiepileptic Medications can be affected by different factors including; individual patient factors (demographic and socioeconomic features, as well as perception and beliefs about Epilepsy) disease features (seizure frequency and severity), medication use (number of daily doses and side effects), and factors related to patient–provider relationship [30]. In this study about 233 (60%) of the study participants missed at least a dose of their antiepileptic medication. Forgetting followed by run out of medications were found to be the major reasons for non-adherence.

Being younger age was reported as a determinant factor for willing to receive medication reminder messages. A study on RVI patients in Northwest Ethiopia indicated that those patients who were 15–30 years of age were 5.18 times more likely to be willing to receive medication reminders [26]. Other studies also indicated that being younger (18–35 years) were more likely to show interest in receiving text message reminders [29, 31]. In contrast, our findings indicated that being older (aged > 40 years) were more willing to receive medication reminder messages, however it was not significant. This might be due to the fact that forgetfulness is one of the side effects of antiepileptic medications and it’s more common in the elderly. In this study, forgetfulness was more commonly experienced by those above 40 years of age and it was significantly associated with willingness to receive medication reminder.

In this study, the result shown that; marital status other than single, urban settlement and higher average monthly income (> 2000 birr) were significant determinants for willingness to receive medication reminder messages. However, no demographic characteristics that predicted a patient’s interest in receiving electronic medication reminders were reported among psychotic patients in UK [29]**.**

This study also found out that, having open communication with the healthcare provider (AOR = 4.13), getting the necessary education or assistance during healthcare visit (AOR = 4.01), experience of forgetting things since they started AED (AOR = 3.26), and not already utilizing other medication reminders (AOR = 4.23) were strong determinant factors for willing to receive medication reminder messages. Ever missing a healthcare appointment (AOR = 2.91, *P* = 0.09) and forgetting to take AED (AOR = 3.06, *P* = 0.09) showed a weak statistical association with interest in receiving text message reminders. The findings of this study implied that there is a huge potential to implement and integrate cell-phone based reminder systems to increase treatment adherence in epileptic patients in developing countries particularly in Ethiopia. The study also indicated that there will be implementation related issues that needs to be considered for future scale up like switching of cell-phones and phone sharing to others.

The main limitation of this study could be an interviewer and social desirability bias since data was collected with interviewer administered technique. The selected study participants may not represent the population in the study area. The questionnaire may not cover all the patients’ concerns on the use of cell phone.

## Conclusion

The findings of this study showed that willingness to use cell phone for AED reminders is high. Marital status, place of residence, average monthly income, experience of forgetting things since they started AED, forget to take AED are the most notable factors that are associated with the willingness of patients to receive cell phone medication reminder messages. Based on this result, implementing a cell phone-based health message reminders for adherence support, counselling and interventions targeting to improve the knowledge of patients to solve problem areas in epilepsy might change the livelihood of patients with epilepsy.

## Data Availability

All major data have been presented in the manuscript.

## Abbreviations

AEDs: Antiepileptic Drugs
AOR: Adjusted Odds Ratio
CNS: Central Nervous System
OR: Odds Ratio
RVI: Retro Viral Infection
